# 2012 and 2013 Peer Review Panel

**DOI:** 10.3109/02813432.2013.847137

**Published:** 2013-12

The Editor-in-Chief and the Editorial Board would like to thank the following professionals who provided peer review for the Scandinavian Journal of Primary Health Care manuscripts in 2012 and 2013:

Steen Z Abildstrøm

Denmark

Lars Agréus

Sweden

Jette Ahrensberg

Denmark

John Sahl Andersen

Denmark

Stig J Andersson

Sweden

Malin André

Sweden

Sibyl Anthierens

Belgium

Michael Bech

Denmark

Bodil Hammer Bech

Denmark

Calle Bengtsson

Sweden

Niels Bentzen

Norway

Mari Bjorkman

Norway

Brian Bjørn

Denmark

Trine Bjørner

Norway

Hördur Björnsson

Iceland

Gunnar Tschudi Bondevik

Norway

Søren Brage

Norway

Mette Brekke

Norway

Trine Brogaard

Denmark

Lucy Brookes-Howell

United Kingdom

Lars Carlsson

Sweden

Kaj Sparle Christensen

Denmark

Morten Bondo Christensen

Denmark

Rene dePont Christensen

Denmark

Niels Damsbo

Denmark

Annette Sofie Davidsen

Denmark

Ynse de Boer

Denmark

Casper den Heijer

The Netherlands

Robert Eggertsen

Sweden

Anders Ekedahl

Sweden

Monika Engblom

Sweden

Lars Englund

Sweden

Arne Fetveit

Norway

Lindsay Forbes

United Kingdom

Annika Forssén

Sweden

George Kenneth Freeman

United Kingdom

Jan C. Frich

Norway

Karin Frydenberg

Norway

Anne Frølich

Denmark

Oddvar Førland

Norway

Linn Getz

Norway

Birgitta Grahn

Sweden

Anette Graungaard

Denmark

Tore Gude

Norway

Mai-Britt Guldin

Denmark

Kjell Hermann Halvorsen

Norway

William Hamilton

United Kingdom

Kjell Haug

Norway

Arja Helin-Salmivaara

Finland

Stefan Heytens

Belgium

Liisa Annikki Hiltunen

Finland

Per Hjerpe

Sweden

Per Hjortdahl

Norway

Stefan Hjørleifsson

Norway

Jens-Christian Holm

Denmark

Tomas Holm

Denmark

Pekka Olavi Honkanen

Finland

Hannes Hrafnkelsson

Iceland

Linda Huibers

Denmark

Eva Hummers-Pradier

Germany

Steinar Hunskaar

Norway

Niels Høiby

Denmark

Bibi Hølge-Hazelton

Denmark

Ole Rikard Haavet

Norway

Dorte Jarbøl

Denmark

Lars Jerden

Sweden

Harald Jodalen

Norway

Melvyn Jones

United Kingdom

Jón Steinar Jónsson

Iceland

Thomas Kanter

Sweden

Svein Kjosavik

Norway

Niels Kristian Kjær

Denmark

Henrikje Klasen

The Netherlands

Thomas Bøllingtoft Knudsen

Denmark

Antti Koivukangas

Finland

Tuomas-Heikki Koskela

Finland

Elise Kosunen

Finland

Allan Krasnik

Denmark

Svend Kreiner

Denmark

Lasse T. Krogsbøll

Denmark

Linda Kærlev

Denmark

Per Lagerlov

Norway

Torsten Lauritzen

Denmark

Laurent Letrilliart

France

Jinhu Li

Australia

Elisabet Londos

Sweden

Jørgen Lous

Denmark

Peter Lucassen

The Netherlands

Kirsten Lykke

Denmark

Monica Britt Löfvander

Sweden

Helle T Maindal

Denmark

Bertil Marklund

Sweden

Harm van Marwijk

The Netherlands

Eivind Meland

Norway

Patrik Midlöv

Sweden

Thomas Mildestvedt

Norway

Richard Morriss

United Kingdom

Pekka Mäntyselkä

Finland

Jörgen Månsson

Sweden

Anna Nager

Sweden

Mette Asbjørn Neergaard

Denmark

Mikko Nenonen

Finland

Maarit Nevalainen

Finland

Anni Brit Sternhagen Nielsen

Denmark

Peter M Nilsson

Sweden

Carsten Obel

Denmark

Nanna J Olsen

Denmark

Louise Olsson

Sweden

Teresa Pawlikowska

United Kingdom

Anette Fischer Pedersen

Denmark

Lars-Göran Persson

Sweden

Christer Petersson

Sweden

Halfdan Petursson

Norway

Guttorm Raknes

Norway

Charlotte Rask

Denmark

Kari Reijula

Finland

Harald Reiso

Norway

Nils Rodhe

Sweden

Sture Rognstad

Norway

Gisle Roksund

Norway

Maria Romøren

Norway

Marianne Rosendal

Denmark

Greg Rubin

United Kingdom

Carl Edvard Rudebeck

Norway

Tony Ryan

United Kingdom

Thomas Salzberger

Austria

Annelli Sandbæk

Denmark

Kaija-Liisa Seppä

Finland

Paul G. Shekelle

USA

Volkert Siersma

Denmark

Hedinn Sigurdsson

Iceland

Jóhann Agust Sigurdsson

Iceland

Jeff Sisler

Canada

Mark Spigt

The Netherlands

Martin Sundqvist

Sweden

Jens Søndergaard

Denmark

Marina Taloyan

Sweden

Gunnar Tellnes

Norway

Alan Tennant

United Kingdom

Ajay Thapar

United Kingdom

Geir Thue

Norway

Torill Tveito

Norway

Stig Valdemarsson

Sweden

Chris van Weel

The Netherlands

Roderick PVenekamp

The Netherlands

Peter FM Verhaak

The Netherlands

Rolf Wahlström

Sweden

Frans Boch Waldorff

Denmark

Thorne Wallman

Sweden

David PB Watson

United Kingdom

Patrik Wennberg

Sweden

Erik L. Werner

Norway

Søren Dinesen Østergaard

Denmark

Ivar J. Aaraas

Norway

